# Evaluation of effectiveness of the using a pillow in children for dental rehabilitation under general anesthesia in term for facilitate intubation

**DOI:** 10.12669/pjms.321.8956

**Published:** 2016

**Authors:** Dilek Günay Canpolat, Mustafa Denizhan Yıldırım, Kenan Cantekin, Aynur Aki

**Affiliations:** 1 Dilek Günay Canpolat, MD, Assistant Professor in Anesthesiology, Department of Oral and Maxillofacial Surgery, Erciyes University Faculty of Dentistry, Kayseri, Turkey; 2 Mustafa Denizhan Yıldırım, MD, Assistant Professor in Anesthesiology, Department of Pediatric Dentistry, Erciyes University Faculty of Dentistry, Kayseri, Turkey; 3 Kenan Cantekin, DDS, PhD, Assistant Professor, Department of Pediatric Dentistry, Erciyes University Faculty of Dentistry, Kayseri, Turkey; 4 Aynur Akin, MD Professor, Department of Anesthesiology, Erciyes University Faculty of Medicine, Kayseri, Turkey

**Keywords:** Child, Intubation, Pillow

## Abstract

**Background and Objective:**

Airway safety may be provided with endotracheal intubation especially for oral procedures because of some potential risks such as aspiration of secretion or foreign bodies. In this study, we aimed to determine whether placing a pillow under the occiput may facilitate endotracheal intubation in non-cooperative children whose extensive dental treatments were planned to take place under general anesthesia.

**Methods:**

The study was performed in Erciyes University, Faculty of Dentistry between March-July 2014. A total 150 ASA I-II children, between 3-9 years were included in this study. Pillow was folded under the occiput in Group 1 (n=75), patients lay on a flat surface in Group 2 (n=75) during the anesthesia induction and intubation period.

**Results:**

There were no statistically significant differences between groups regarding the demographic data (age, weight, gender) (p>0.05). Operation times were similar in both groups (p=0.329). The number of intubation attempts was smilar in both groups (p=0.412). The intubation time was longer in group one than in group two (p= 0.025).

**Conclusion:**

We concluded that, placing a pillow under the patients occiput provided longer intubation time without changing the number of attempts in the normal airway in non-cooperative children whose extensive dental treatments were planned to take place under general anesthesia.

## INTRODUCTION

Dental problems in children can be generally solved under local anesthesia by pediatric dentists with non-pharmacological methods. However, this is not always possible for non-cooperative pediatric patients with severe anxiety or mental retardation. Therefore, dental treatment in non-cooperative children may require conscious sedation with nitrous oxide/oxygen, deep sedation or general anesthesia.^1^ If children have extensive problems and can not cooperate for several reasons, general anesthesia may be unavoidable.^2^ Airway safety may be provided with endotracheal intubation especially for oral procedures because of potential risks such as aspiration of secretion or foreign bodies. Infants and young children in particular have increased risk of aspiration because of certain own anatomic and physiological features. Oral procedures may lead to further increase in aspiration risk. A good airway management technique is essential and may help prevent the undesirable adverse effects of tracheal intubation.^3^

Endotracheal intubation may facilitate by correct positioning of the head. The ‘siniffing position’ has been generally advocated as the standard position faciliating direct laryngoscopy and appears to be advantageous. It is also suggested to optimized for external laryngeal pressure, changing laryngoscope blades and repositioning to patient’s head and neck too.^4^ The siniffing position provide aproximately 35° of flexion of the lower cervical spine on the chest and extention at the atlanto-occipital joint. In another expression, the optimal angle for neck flexion was determined as 35° and face extension as 15°. The sniffing position could be achived by elevating the head by a pillow or pads under the occiput. However this is not always adequet for intubation condition. Using an uncompressible pillow may provide a beter sniffing position for intubation in adults.^5^,^6^ The optimal head positioning for laryngoscopy is a much debated controversial subject especially in anesthetized children. Some authors have suggested slightly extending the patients position by elevation of the occiput in children. On the other hand, others have suggested resting position to facilitate visualization of the glottis. El-Orbany et al^5^ did not suggest head elevation in infants and small children because according to their theory the size and shape of the head allow axis approximation in the head-flat position.

The main objective of this study was to determine whether using a pillow under the patients occiput may facilitate endotracheal intubation in non-cooperative children whose extensive dental treatments were planned to take place under general anesthesia.

## METHODS

The study protocol was approved by the Local Ethics Committee of Erciyes University, and was performed in Erciyes University, Faculty of Dentistry between March-July 2014. A written consents from the parents were obtained. One hundred and fifty ASA I-II pediatric patients, between the ages of 3-9 years, who were admitted to the Erciyes University, Faculty of Dentistry, were included in this study. None of the patients were cooperative, they had severe anxiety, mental retardation or disabilty. Because dental procedures could not be performed with conventional methods it was planned to administer under general anesthesia. Patients who had anticipated difficult airway such as those with Apert syndrome, Treacher Collins Syndrome; or with a history of any pathology of the head or neck were not included to the study. Patients with serious respiratory problems, cardiological conditions, renal failure, history of allergic reactions, and patients who had an abnormality in thyromental distance, or mouth opening were also excluded.

All of the children fasted overnight and received EMLA (Eutectic Mixture of Local Anesthetics: Astrazeneca, London, UK) cream treatment for vascular access, unless contraindicated, and were pre-medicated using intravenous midazolam before being taken to the operating room. A noninvasive standard monitoring procedure was applied in all cases during the procedure. Patients were randomly divided into two groups. Intravenous induction was applied to all children with 2 mg/kg propofol and 0.6 mg/kg rocuronium as neuromuscular relaxant. Ventilation was supplied by mask until endotracheal intubation. Ventilation was controlled to maintain a PaCO_2_ pressure of 35-40 mmHg. Intravenous infusion of 2.5 glucose with 70 mm sodium was applied at a rate of 5 ml/kg/h intraoperatively and 3 ml/kg/h postoperatively until oral intake. The residual neuromuscular blockade was reversed with 0.02 mg/kg atropine and 0.04 mg/kg neostigmine intravenously.

Intubation was performed by two anesthesiologists who had a minimum professional experience of 4 years. A pillow was folded under the occiput in Group 1 (n=75), the patients were laid on a flat surface in Group 2 (n=75) during the anesthesia induction and intubation period. Direct laryngoscopy was performed in all patients by an appropriately sized Macintosh blade. Intubation time and number of attempts, need for stylet, Cormack Lehane scores that evaluate glottic visualization during laryngoscopy [7], thyromental distance, mouth opening and neck movement normality and operation time were recorded. Also endotracheal tube and blade size, oral airway need for maintaining ventilation, burp maneuver requirement, and number of attempts were recorded. Electrocardiogram (EKG), heart rate (HR), mean artery pressure (MAP), peripheral oxygen saturation (SpO_2_) were documented before and after intubation; fifth and tenth minutes, before and after the extubation also. Postoperatively complications such as bronchospasm, laryngospasm, reintubation, hypoxia, laryngeal edema, racemic epinephrine need, cold vapor application and sore throat were recorded.

### Statistical analysis

The independent t-test was used to compare intergroup differences in demographic and clinical variables. Statistical analyses were performed using the Statistical Package for the Social Sciences (SPSS), version 15.0 (SPSS Inc., Chicago, IL, USA) for Windows.

## RESULTS

There were no statistically significant differences between groups regarding the demographic data (age, weight, gender) (p>0.05, Table-I). No significant differences were found between groups regarding HR, MAP and SPO_2_ values at any times (p>0.05, Table-II). Cormack Lehane scores were similar in both groups (p=0.41). There were no statistically significant differences between groups regarding endotracheal tube and blade size, stylet or oral airway need for intubation, or BURP maneuver requirement (p>0.05). Operation times were similar in both groups (p=0.329, Table-I). Although, the number of attempts was similar in both groups (p=0.412, Table-I), the intubation time was longer in group 1(p= 0.025, Table-I). On the other hand, there were no statistically significant differences regarding adverse effects such as trauma, bronchospasm, cold vapor application or sore throat (p>0.05, Table-III). Laryngospasm, reintubation, hypoxia, laryngeal edema, racemic epinephrine need were not observed in any of the patients.

**Table-I:** The demographic data, operation time, intubation time, number of attemps of the groups.

	Group 1 (n=75) (X±SD)	Group 2 (n=75) (X±SD)	P
Age (year)	5.5±1.6	5.8±2.2	0.388
Gender (M/F)	48/27	48/27	1.00
Weight (kg)	20±5.3	20±2.2	0.527
Operation time (min)	79±19.7	83±32	0.329
Intubation time (seconds)	18,8±14,5	14,4±0,41	0.025*
Number of attemps	1.2±0.56	1.17±0.41	0.412

* Intubation time was longer in Group 1

**Table-II:** Hearth Rate (HR), Mean Arterial Pressure (MAP), SPO_2_ values.

HR (beat/min)/MAP (mmHg)/SPO_2_ (%)	Group 1 (X±SD)	Group 2 (X±SD)	p
Before the intubation	82.2±12.3/ 109±13.8/ 98.7±2.9	89.9±11.6/ 106±17.9/ 99.7±4.8	0.386/0.213/0.11
After the intubation	92±15.6/ 121±43.2/ 99.9±2	91.4±13.8/ 130±14/ 99.3±1.15	0.791/0.593/0.38
5. min	81±11.3/ 115±16/ 98.5±10.3	83.2±12.8/ 113±16/ 99.7±0.6	0.274/0.662/0.303
10. min	73.1±10.03/ 108±19/ 99.6±0.6	73.7±10.5/ 110±15/ 99.7±7	0.734/0.497/0.386
Before the extubation	76.7±13.4/ 102±13/ 99.4±0.9	76.8±13.1/ 103±15/ 99.7±0.6	0.932/0.342/0.272
After the extubation	89.3±13.8/ 101±15/ 99.4±0.9	85.3±12.3/ 127±11/ 99.6±0.6	0.34/0.06/0.09

**Table-III:** Adverse effects in groups.

	Group 1	Group 2	p
Broncospasm	5 patient	2	0.335
Trauma	1	-	0.319
Cold vapor application	10	7	0.101
Sore throat	10	13	0.173

## DISCUSSION

The main findings of our study is that placing a pillow under the occiput provided longer intubation time without changing the number of attempts in the normal airway in non-cooperative children whose extensive dental treatments were planned to take place under general anesthesia. Therefore the neutral position was better in children for endotracheal intubation.

In general anesthesia, endotracheal intubation provides airway safety for oral procedures. Children’s airway anatomy has some differences than adults and there is a lack of guidelines based on scientific evidence on airway management in terms of head positioning in children. The position’s effects on the airway are qualitative rather than qualitative in children. In practice, pediatric anesthesiologists interests in some adults studies because of limited knowledge about pediatric posture and airway management for intubation. However, pediatric anesthetists and intensivists are concerned about optimal glottic visulaization and try to facilitate intubation by using some techniques such as head positioning.^8^ The age of children is the most important factor of head posture.^9^ The sniffing position can be ensured by placing a pillow under the occiput of the patient and results in atlanto-occipital extension and cervical flexion.^10^ Thus the neck is flexed and the head is extended by the sniffing position. Adnet et al.^11^ used a cushion under the neck of the adult patients to provide the sniffing position to determine its effect on intubation before the anesthesia. They reported that this positioning was advantageous only for obese or head-extentsion-limited patients in adults. In another study, Adnet et al.^12^ used magnetic resonance imaging and observed no alignment of the laryngeal, pharyngeal or mouth axes in awake patients with normal airway anatomy. In contrast, Vialet et al. 8 confirmed that in infants and young children, a slight head extension at 190 and 130 improves the vision of the glottis and laryngeal axis. In the present study we used a pillow under the occiput to determine whether it facilitate the intubation or not. Thus, we achieved a slight cervical flexion by using an uncompressible pillow. We observed ın our study that, in the pillow group the intubation time was longer but the pillow did not affect the number of attempts in children’s normal airway.

Schmitt et al.^13^ reported that elevation of the head and neck and external laryngeal pressure may cause better visualization of glottic structures than the sniffing position. Takahata et al.^14^ suggested the BURP maneuver to provide better visualization of the larynx. In our study, to improve visualization of the larynx, the BURP maneuver was used when necessary. However, the pillow did not change the requirement for the BURP maneuver. When children lie on a flat surface, the neck becomes flexed and this situation may cause airway obstruction especially in the anesthesia induction period. An oral airway may improve ventilation with a face mask in lateral positioning and relieve obstruction.^15^ We performed intubation with appropriate Macintosh blade according to the age of patients. Intubation tube sizes were similar for both groups. These provided standardization between the groups. Thus the intubation conditions were minimally affected by these possible affecting factors.

The anatomical differences in children may lead to more airway obstruction under sedation or general anesthesia than in adults.^3^ Therefore, ventilation in children requires attention and may not be easy. Paal et al.^16^ reported a study which focused on head position angles in children for opening the upper airway. They suggested the use of the neutral head position in pre-school children (1-5 years) with an angle of 1^0^ and 13^0^, head extension of 16^0^ in school children (6-10 years) to achieve optimal ventilation. In our study the children were between the age of 3-9 years as in Paal et al.’s pre-school group. We randomly divided the patients into two groups. We observed that pillow usage did not change the oral airway need for ventilation however it faciliated ventilation by opening the upper airway. In this present study, patients were kept in a supine position during intubation and surgery. We observed that endotracheal intubation was easy in this neutral position. In addition, placing a pillow under the occiput of children caused longer intubation time. According to our experience, putting a pillow under the occiput supplied atlanto-occipital extension and cervical flexion. This anatomic modification may lead to longer intubation time in children’s normal airway.

Intubation difficulty may be evaluated with Cormack Lehane scores.^7^ Mallampati clasification requires patient compliance with the anesthesiologist for physical examination.^17^ This is because the patient is required to open the mouth and put the tongue out simultaneously for Mallampati score evaluation. This examination may be performed in adults easily but it is always not possible in smaller children. In the present study, Mallampati scores could not be evaluated in all patients because some of them were under three years old and did not cooperate in this examination. However, Cormack Lehane scores determine glottic appearance during laryngoscopy. In our study, there were no differences in Cormack Lehane scores between the groups because the study was designed in the normal airway in children. Several physical examination findings indicate that difficult airway in adults may also apply to children. Also thyromental distance, head and neck movement,^18^ and the upper lip bite test,^19^ have been described in adults for determining difficult airway, but they may not be applied in children. In our study, thyromental distance, head and neck movement and mouth opening were normal in physical examination.

### Limitations of the Study

We did not calculate the values with a standard measure method. This was one of the limitations of our study. The other limitation was the wide range in the age of patients. The ages of patients can affect intubation procedure, and side effects. Nonetheless, on the positive side, only fifeteen patients were under three years of age.

During laryngoscopy and endotracheal intubation, a cardiovascular response may occur with stimuli. Several medical^20^ and technical methods have been used to supress it. Haidry et al.^21^ compared the effect of McCoy and Macintosh blade on hemodynamic response and concluded the McCoy laryngoscope was better with shorter duration. In our study we used Macintosh blade for all patients and there was no differences the blade size. Further more the intubation technique may be used to affect the hemodynamic response to laryngoscopy. In our study we used two different methods by using a pillow in one group and not using in the other group. However, the hemodynamic response was similar in both groups and there were no differences in MAP and HR in both groups; before and after intubation, fifth and tenth minutes, before and after extubation or in SPO_2_ values as. Therefore, we can say that using a pilow did not affect the hemodynamic response to intubation in pediatric patients’ normal airway.

Although, endotracheal intubation is commonly performed, it may cause some undesirable side effects. It may lead to injury of soft tissue throughout the respiratory tract such as dental injury or mucosal lacerations.^22^ In the present study trauma was observed in only one patient in the pillow group. A retrospective study showed that airway complications such as stridor, laryngospasm, desaturation salivation had a high incidence in children.^23^ In this study, bronchospasm was seen in 5 patients in group one whereas it was seen in two patient in group two. Also sore throat and cold vapor need were observed in both groups but this was not statistically important. Although endotracheal intubation provides safety airway in dental procedures, it is often accompanied with airway complications when compared with laryngeal mask.^24^ If intubation time is longer or the procedure is difficult, it is possible to encounter more airway complications. In the present study, although intubation duration was longer in the pillow group, there were no significant differences with regard to side effects.

In conclusion, we found that, placing a pillow under the patient’s occiput provided longer intubation time without changing the number of attempts in the normal airway in non-cooperative children and neutral positioning was better in these patients.
